# Risk factors for progression of radiographic knee osteoarthritis in elderly community residents in Korea

**DOI:** 10.1186/s12891-018-1999-5

**Published:** 2018-03-12

**Authors:** Jong Jin Yoo, Dong Hyun Kim, Hyun Ah Kim

**Affiliations:** 1 Department of Internal Medicine, Kangdong Sacred Heart Hospital, Seoul, South Korea; 2 Department of Social and Preventive Medicine, Hallym University College of Medicine, Chuncheon, South Korea; 3 Rheumatology Division, Department of Internal Medicine, Hallym University Sacred Heart Hospital, 896, Pyongchondong, Dongan-gu, Anyang, Kyunggi-do 431-070 South Korea

**Keywords:** Osteoarthritis, Knee, Risk factors, Progression

## Abstract

**Background:**

Knee osteoarthritis (OA) is the most common form of arthritis affecting the elderly. Understanding the risk factors for knee OA has been derived from cross sectional studies. There have been few longitudinal studies of risk factors for knee OA among Asian populations. The purpose of this study was to evaluate the risk factors for knee OA in elderly Korean community residents.

**Methods:**

This prospective, population-based study was conducted on residents over 50 years of age in Chuncheon who participated in the Hallym Aging Study. Standardized weight-bearing semi-flexed knee anteroposterior radiographs were obtained in 2007 and in 2010. Of 504 participants at baseline, 322 participants (male: female = 150:172) underwent follow-up knee radiographs. Radiographic knee OA was defined as Kellgren/Lawrence (K-L) grade of ≥ 2. Risk factors assessed at baseline were tested for their association with incidence, progression, and worsening of radiographic knee OA by logistic regression analysis.

**Results:**

The median age of these participants at follow-up was 71 years **(**interquartile range 66–75 years). Incident OA was observed in 33 (10.2%) and progression of OA (defined as an increase of Kellgren-Lawrence (K-L) grade at follow-up, from grades 2 or 3 at baseline) in 43 (13.55%) participants. In multivariate logistic regression analysis, only females were significantly associated with the progression of radiographic knee OA (odds ratio [OR] = 4.41, 95% confidence interval [CI] 1.32–14.77).

**Conclusions:**

In this 3-year longitudinal study, the yearly incidence and progression of knee OA was higher than those previously reported in Western populations.

## Background

Knee osteoarthritis (OA) is the most common form of arthritis affecting the elderly and is a growing public health concern as the population ages. In the US, in 2004, approximately 431,485 primary knee replacements were performed [1]. This was a 53% increase in primary knee replacements, compared with data from 2000. From 2002 to 2005, 103,601 total knee replacement (TKR) surgeries were performed in South Korea, and approximately 83% of these were associated with knee OA [2]. The rate of TKR increased over the 4 years of the study and was much higher in women than in men. In rapidly aging societies such as in Korea, the increasing prevalence of knee OA may present serious new health issues. Previous studies have reported various risk factors associated with knee OA such as older age, female sex, hypertension, raised glucose, obesity, history of knee injury, varus/valgus malalignment, quadriceps muscle strength, and physical workload [3–12]. However most of these studies for risk factors of knee OA have been performed in persons of European origin, so the results cannot be extrapolated to Asian populations. There have only been a few longitudinal studies of risk factors for knee OA among Asian peoples [13, 14]. We have previously examined the prevalence of radiographic knee OA (ROA) and symptomatic OA in a 2007 cross-sectional study, using the standardized radiographic protocol, and the prevalence was 37.3% and 24.2%, respectively. The presence of hypertension, having a manual occupation and a lower level of education were significantly associated with the presence of ROA [15]. However, cross-sectional studies can neither show how risk factors affect the progression of knee OA, nor define the cause and effect relationship. Therefore, longitudinal studies are needed to clarify the risk factors for the incidence or the progression of knee OA. The objective of the present study was to assess the incidence, progression, and worsening of radiographic knee OA in elderly Korean community residents during a 3-year follow-up period and, furthermore, to evaluate the prospective risk factors for knee OA.

## Methods

### Participants

The participants in this study were recruited in the Hallym Aging Study (HAS), which commenced in 2004 and involved follow-up examinations at 3-year intervals. The HAS is a prospective cohort of residents aged 50 years or older (70% older than 65 years) in Chuncheon, a city in the northeast area of South Korea. Details of the cohort profile were reported elsewhere [15] and are only briefly described here. The city was divided into 1408 areas based on the Korean National Census conducted in 2000, and 200 areas were randomly selected [16]. Nine hundred eighteen of the 1489 participants completed face to-face interviews at baseline in 2004. Of the 918 participants, 702 participated in the 2007 survey, excluding 216 of them who died, moved, refused participation, or could not be contacted. Among the 702 participants, 504 who underwent knee radiography participated in the 2007 OA study cohort. After 3 years, 182 patients were lost to follow-up and 322 completed the survey, including radiographs, and constituted our present 2010 study cohort. The Hallym University’s institutional review board approved the study protocol, and informed consent was obtained from all the study participants.

### Data collection

Demographic information, such as educational level, marital status, income, occupation, regular exercise, and comorbidities was collected through face-to-face interviews by trained interviewers. Educational levels were classified as < 10 or ≥10 years. Income was divided into 11 categories, and low income was defined as < 500,000 Korean Won (1000 Korean won is approximately 1.00 US dollars) per month. Occupations were categorized as follows: none, mostly sedentary work, work demanding some walking, work demanding physical exertion, and work demanding heavy physical exertion. Manual work was defined as work demanding physical or heavy physical exertion. Exercise status was self-reported, and answers were classified as < 3 times/week or ≥3 times/week. Smoking was defined as more than 20 packs of cigarettes smoked during the participants’ lifetime. Alcohol consumption was defined as the drinking of any alcoholic beverage more than once per month. Comorbidity health information was also self-reported and recorded using 29 predefined diagnostic categories, which included hypertension, diabetes mellitus, arthritis, stroke, and osteoporosis. Body mass indexes (BMIs) were calculated as the body weight divided by the height squared (kg/m^2^).

### Radiographic assessment

All the participants underwent radiographic examination of both knees in a weight-bearing anteroposterior view with a semi-flexed knee position. A Plexiglas frame (SYNARC, San Francisco, CA, USA) was used to standardize the knee positions. Details of the study protocol were described elsewhere [15]. Knee OA severity was classified as grade 0–4 according to the Kellgren/Lawrence (K-L) grading system. Radiographic OA was defined as a K-L grade of ≥ 2, and severe radiographic OA was defined as a K-L grade of 3 or 4. Radiographs were read twice by one reader, an academically-based rheumatologist of 17 years of experience (HAK). The reproducibility of the intra-reader assessments was high (for OA vs. no OA, κ = 0.89). Films that allocated different K-L grades at the two readings were adjudicated through consensus between the original reader and a second reader (David Hunter at the University of Sydney).

### Statistical analysis

The participants were divided into 4 age groups, namely, 50–59 (29 participants), 60–69 (88 participants), 70–79 (178 participants), and 80–89 years (27 participants). Due to the inherent limitations of complete case analysis, a post hoc available-case analysis was performed, when possible, to check for dropout bias. The age-specific prevalence of 3-year incidence, progression, and worsening of radiographic knee OA were calculated. The incidence of radiographic knee OA was defined as having a K-L grade of 0 or 1 at baseline and a grade of ≥ 2 (radiographic OA) at follow-up. Progression was defined as an increase of the K-L grade at follow-up from grades 2 or 3 at baseline. Worsening was defined as an increase in the K-L grade at follow-up from any other grade (including grades 0 and 1). The group with worsening knee OA essentially included incident cases. The annual cumulative incidence, progression, and worsening were calculated by dividing them with the number of years under observation. To compare participants with/without OA, continuous variables were tested using the Mann-Whitney U test, and categorical variables were tested using Fisher’s exact test. Crude odds ratios (OR) for risk factors for incidence, progression, and worsening of radiographic knee OA were calculated using 95% confidence intervals (CI). Adjusted ORs were calculated using logistic regression analysis after adjusting for the factors significantly associated with incidence, progression, and worsening of knee OA in univariate analysis. Data were analyzed using SPSS version 15. Data are presented as median and interquartile ranges (IQR) or as percentages. *P* values < 0.05 (2-tailed) were considered statistically significant.

## Results

### Characteristics of the study participants

Of the 504 participants who underwent knee radiographs in the 2007 survey, 322 completed the survey, including radiographs, and constituted our 2010 study cohort. There was no significant difference in age and sex between the complete follow-up group and the group lost to follow-up (Table 1). The median participant age was 71.0 years, and 53.4% were women in the complete follow-up group. Fifty-eight participants (18%) had moderate to severe OA, defined as a K-L grade of ≥ 3. The characteristics of the 504 participants at baseline in this study are shown in Table 1. The median age of subjects with knee OA was higher than those without knee OA (72.64: 68.62 years) (Table 2).

**Table 1:** Baseline characteristics of the entire cohort, participants with complete follow-up, and participants lost to follow-up

^a^Characteristics	Entire cohort (*n* = 504)	Complete follow-up (*n* = 322)	Lost to follow-up (*n* = 182)
Age, median (IQR) years	71 (66.0–75.0)	71.0 (66.0–75.0)	72 (65.0–76.0)
Women	54.4	53.4	56.0
BMI, median (IQR) kg/m^2^	24.7 (22.4–26.7)	24.6 (22.4–26.5)	25.2 (22.4–27.0)
Lower level of education	78.0	75.5	82.4
Low income	24.4	22.0	28.6
Exercise (≥ 3 times/week)	26.0	28.3	22.0
Previous or current smoker	40.5	40.7	40.1
Previous or current alcohol consumption	41.5	39.4	45.1
Manual occupation	19.8	21.4	17
K/L grade in worst knee			
Grade 3	9.7	9.6	9.9
Grade 4	9.3	8.4	11.0
Diabetes mellitus	10.1	9.0	12.1
Osteoporosis	19.2	19.3	19.2

*IQR* interquartile range, *BMI* body mass index, *K-L* Kellgren-Lawrence, *TKR* total knee replacement

^a^Except where indicated otherwise, values are written as percentages. Levels of education were classified as < 10 years or ≥ 10 years. Income was divided into 11 categories and low income was defined as < 500,000 Korean won per month. Exercise status was self-reported and responses were classified as < 3 times/week or ≥ 3 times/week. Smoking was defined as more than 20 packs of cigarettes having ever been smoked during the participants’ lifetime. Alcohol consumption was defined as the drinking of any alcoholic beverage more than once per month. Manual work was defined as work demanding physical or heavy physical exertion. Co-morbidity health information was also self-reported, and was recorded using 29 pre-defined diagnostic categories. Diabetes mellitus was defined as either a fasting glucose level ≥ 126 mg/dL or a 2-h glucose level of ≥200 mg/dL after 75-g oral glucose loading, or treatment for previously diagnosed diabetes mellitus

**Table 2:** Baseline characteristics of the subjects with/without knee osteoarthritis

^a^Characteristics	No. of subjects	No knee osteoarthritis (*n* = 307, 60.9%)	Knee osteoarthritis (*n* = 197, 39.1%)	*P* value
Age, median (IQR) years		68.62 (67.70–69.53)	72.64 (71.67–73.62)	< 0.001
Sex				< 0.001
Men	230	84.3	15.7	
Women	274	41.2	58.8	
BMI kg/m^2^				0.001
< 25	264	67.4	32.6	
≥ 25	239	53.6	46.4	
Lower level of education	393	53.4	46.6	< 0.001
Low income	123	49.6	50.4	< 0.001
Exercise (≥ 3 times/week)	131	75.6	24.4	< 0.001
Previous or current smoker	204	81.9	18.1	< 0.001
Previous or current alcohol consumption	209	76.1	23.9	< 0.001
Manual occupation	100	32	68	< 0.001
Marriage (living without spouse)	157	36.3	63.7	< 0.001
Diabetes mellitus	51	60.8	39.2	0.984
Osteoporosis	97	44.3	55.7	< 0.001

*IQR* interquartile range, *BMI* body mass index

^a^Except where indicated otherwise, values are written as percentages. Levels of education were classified as < 10 years or ≥10 years. Income was divided into 11 categories and low income was defined as < 500,000 Korean won per month. Exercise status was self-reported and responses were classified as < 3 times/week or ≥3 times/week. Smoking was defined as more than 20 packs of cigarettes having **e**ver been smoked during the participants’ lifetime. Alcohol consumption was defined as the drinking of any alcoholic beverage more than once per month. Manual work was defined as work demanding physical or heavy physical exertion. Co-morbidity health information was also self-reported, and was recorded using 29 pre-defined diagnostic categories. Diabetes mellitus was defined as either a fasting glucose level ≥ 126 mg/dL or a 2-h glucose level of ≥200 mg/dL after 75-g oral glucose loading, or treatment for previously diagnosed diabetes mellitus

Participants who were not obese (BMI < 25 kg/m^2^) were more likely to have no knee OA (67.4%). The characteristics of the subjects with/without knee OA at baseline are shown in Table 2.

### Prevalence of incidence, progression and worsening of radiographic knee OA

**Fig. 1:**
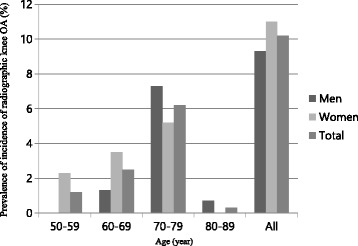
Prevalence of incidence of radiographic knee OA, according to age and sex. Incidence of radiographic knee OA was defined as having a K-L grade of 0 or 1 at baseline and a grade of ≥ 2 at follow-up

**Fig. 2:**
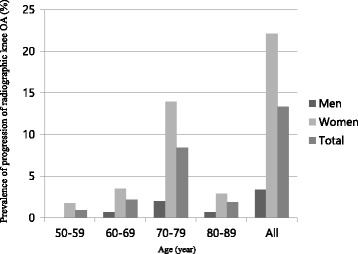
Prevalence of the progression of radiographic knee OA, according to age and sex. Progression of radiographic knee OA was defined as an increase of the K-L grade at follow-up, from grades of 2 and 3 at baseline

**Fig. 3:**
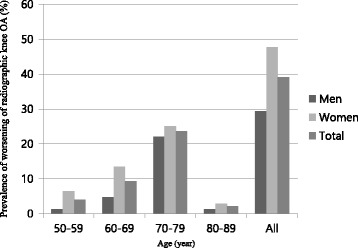
Prevalence of radiographic knee OA worsening, according to age and sex. Worsening of radiographic knee OA was defined as an increase of the K-L grade at follow-up, from any baseline grade

The incidence of radiographic knee OA was observed in 33 (10.2%, male: female [M: F] = 9.3%: 11%) participants (7[2.17%], bilateral), and progression in 43 (13.55%, M: F = 3.33%: 22.09%) participants (15[4.66%], bilateral). The worsening of radiographic knee OA was observed in 126 (39.1%, M: F = 29.3%: 47.7%). The rates of incidence, progression, and worsening were the highest in the 70–79 age groups (6.2%, 8.39%, 23.6%, respectively), and leveled off afterwards. Women tended to have higher rates of progression and worsening in all age groups. The prevalence of incidence, progression, and worsening of radiographic knee OA in respect of age and sex are summarized in Figs. 1, 2, and 3.

### Longitudinal risk factors for radiographic knee OA

We analyzed the data to determine risk factors for the progression of radiographic knee OA (Table 3). In the univariate analysis, sex, smoking, alcohol consumption, manual occupation, marriage, education level and osteoporosis were significantly associated with the progression of radiographic knee OA. However, in the multivariate logistic regression analysis, only women were significantly associated with the progression of radiographic knee OA (OR = 4.41, 95% CI 1.32–14.77). We next performed an analysis to determine the risk factors for worsening of radiographic knee OA (Table 3). Being female (OR = 1.41, 95% CI 1.02–1.95), and having a lower level of education (OR = 0.52, 95% CI 0.35–0.77) were significantly associated with a worsening of radiographic knee OA in the univariate analysis. In the multivariate logistic regression analysis, only a lower level of education was significantly associated with worsening of radiographic knee OA (OR = 0.56, 95% CI 0.37–0.86). In the incidence analysis of radiographic knee OA, we could not find any correlating risk factor.

**Table 3:** Risk factors for progression and worsening of radiographic knee osteoarthritis in elderly community residents

	Progression of knee OA		Worsening of knee OA	
	Crude OR (95% CI)	Adjusted OR (95% CI)	Crude OR (95% CI)	Adjusted OR (95% CI)
Age	1.04 (0.99–1.09)		0.99 (0.98–1.02)	
Sex				
Men	1.0	1.0	1.0	1.0
Women	7.25 (2.80–18.74)	4.41 (1.32–14.77)	1.41 (1.02–1.95)	1.21 (0.85–1.71)
BMI, kg/m^2^				
< 25	1.0		1.0	
≥ 25	1.44 (0.77–2.70)		1.32 (0.95–1.82)	
Education (year)				
< 10	1.0	1.0	1.0	1.0
≥ 10	0.24 (0.07–0.81)	0.59 (0.16–2.10)	0.52 (0.35–0.77)	0.56 (0.37–0.86)
Income (10,000 won/month)				
< 50	1.0		1.0	
50–149	0.85 (0.37–1.94)		0.73 (0.48–1.14)	
≥ 150	0.43 (0.16–1.12)		0.72 (0.47–1.13)	
Exercise				
No	1.0		1.0	
Yes	0.44 (0.18–1.06)		0.71 (0.49–1.03)	
^a^Smoking				
No	1.0		1.0	
Yes (ex-, current)	0.26 (0.11–0.59)		0.79 (0.58–1.11)	
Alcohol consumption				
No	1.0	1.0	1.0	
Yes (ex-, current)	0.29 (0.13–0.65)	0.78 (0.30–2.04)	1.12 (0.81–1.56)	
Manual occupation				
No	1.0	1.0	1.0	
Yes	2.66 (1.37–5.16)	1.74 (0.84–3.58)	0.95 (0.63–1.42)	
Marriage				
Yes (living with spouse)	1.0	1.0	1.0	
No	2.53 (1.35–4.75)	1.11 (0.55–2.26)	1.25 (0.88–1.77)	
Baseline K-L grade				
0	1.0		1.0	
1	1.00 (< 0.001- > 999.99)		0.96 (0.62–1.49)	
2	> 999.9 (< 0.001- > 999.9)		1.21 (0.72–2.03)	
3	> 999.9 (< 0.001- > 999.9)		1.23 (0.66–2.31)	
Diabetes mellitus				
No	1.0		1.0	
Yes	1.19 (0.45–3.17)		1.23 (0.72–2.11)	
Osteoporosis				
No	1.0	1.0	1.0	
Yes	2.48 (1.27–4.84)	1.18 (0.58–2.41)	1.17 (0.77–1.76)	

^a^Most of the women were non-smokers (male: female = 27.3%: 93.6%). Smoking was removed from the multivariate analysis because of the multicollinearity problem with sex

*OR* odds ratio, *95% CI* 95% confidence interval, *BMI* body mass index, *K-L* Kellgren-Lawrence

## Discussion

In this prospective 3-year follow-up study of 504 Chuncheon city residents aged 50 years and older, 322 participants (male: female = 150: 172) underwent a 3-year follow-up knee radiograph. Incidence, progression, and worsening of knee OA were observed in a significant number of participants at the 3-year follow-up. In the multivariate logistic regression analysis, only women were significantly associated with the progression of radiographic knee OA and a lower level of education was significantly associated with the worsening of radiographic knee OA.

A limited number of population-based studies have examined the incidence or progression of radiographic knee OA [8, 13, 14, 17, 18] and only two have reported on Asian populations [13, 14]. In the US Framingham Osteoarthritis Study which involved follow-up after a mean 8.1-year interval, the progression of radiographic knee OA, defined as having a K-L grade of ≥ 2 at baseline and showing an increase of at least one K-L grade at follow-up, was 24.2% and 31.8% (3.0% and 3.9% per year) in men and women, respectively [17]. In the Chingford Women’s Study, a UK community-based cohort were followed-up for more than 14 years, and the annual rates of disease progression and worsening were 2.8% and 3.0%, respectively [18]. In the present study, the annual rate of knee OA progression, and worsening was 7.36%, and 15.9% in women, respectively, which is much higher than that of previous studies in the US and UK [8, 17, 18], implying that progression, and worsening of knee OA is higher among Korean women than in those of European origin. In the Japanese population-based 3-year follow-up ROAD study, the progression rate of knee OA was 6.3% per year in women [14]. The higher progression rate of radiographic knee OA in Korean and Japanese women might be due to lifestyle factors, such as sitting with legs crossed, sitting with knees and feet together on the floor, or genetic factors.

In the Framingham Osteoarthritis Study, the incidence of radiographic knee OA was 1.4% and 2.2% per year, in men and women, respectively [17]. In the Chingford Women’s Study, the incidence was 2.3% per year in women [18]. In the ROAD study, the incidence was 2.0% and 3.7% per year, in Japanese men and women, respectively [14]. In the present study, we also examined the incidence of knee OA, and found that the incidence rate of knee OA was 3.1% and 3.7% per year, in Korean men and women, respectively, which was also higher than that of other previous epidemiologic studies in the US, and the UK [17, 18]. We could not find any risk factors for the incidence of knee OA, which may be attributable to the rather small sample size of the present study.

In this study, only women were significantly associated with the progression of radiographic knee OA after adjustment for covariates including age, BMI, education, income, exercise, smoking, alcohol consumption, manual occupation, marriage, baseline K-L grade, DM, and osteoporosis. Being female has previously been reported as a risk factor for knee OA [6, 13, 14]. Only a low education level was significantly associated with the worsening of radiographic knee OA while being female was significant only in the univariate analysis. The level of education correlates with sex in this study cohort, which suggests that multicollinearity would have been the cause of this discrepancy. Although smoking was negatively associated with the progression of OA in the univariate analysis, it is intuitively improbable that it actually protects against OA progression. In addition, it was strongly correlated with sex; therefore, we excluded smoking in the multivariate analysis. A lower level of education, which was significantly associated with the worsening of radiographic knee OA, has been associated with the increased prevalence, morbidity and mortality of many chronic diseases. Several previous studies have examined the relationship between formal education levels, and hip and knee OA, showing results consistent with our study [19–21]. In the National Health and Nutrition Examination Survey of the USA, adjustment for age, knee injury, ethnicity, obesity, occupation, and low educational attainment were associated with a high prevalence of knee OA in both men and women, [19]. After adjustment for known risk factors, educational attainment, as an indicator of socioeconomic status, is associated with symptomatic knee OA in both men and women and with radiographic knee OA in US women [20]. In a USA study of African-American and European-American men and women aged ≥ 45 years, pain and disability were significantly associated with low educational attainment in radiographic and symptomatic hip OA, after adjusting for covariates included age, sex, ethnicity, BMI, and the presence of knee symptoms [21].

Our study had strengths and limitations. To the best of our knowledge, the present study is the first longitudinal study to evaluate the progression, incidence, and risk factors of radiographic knee OA, using standardized radiographs and a recognized grading system in Korea. However, despite its prospective design, which is rare in Asian population studies, 3 years is a rather short time to evaluate the progression of OA. Our study contains a relatively small sample size, and the previously known risk factors of knee OA may not be statistically significant. The study area included only Chuncheon, a city in South Korea, reducing the representativeness of the study sample.

## Conclusions

The incidence, progression, and worsening of radiographic knee OA were observed in a significant number of participants at 3-year follow-up. Being female was a risk factor for the progression of radiographic knee OA, and having a lower level of education was a risk factor for the worsening of radiographic knee OA in this longitudinal study. Understanding the risk factors for knee OA may provide insights into preventative measures and therapeutic strategies for knee OA.

## Abbreviations

BMI: Body mass index
CI: Confidence interval
DM: Diabetes mellitus
IQR: Interquartile range
K-L: Kellgren/Lawrence
OA: Osteoarthritis
OR: Odds ratio
ROA: Radiographic knee OA
TKR: Total knee replacement
